# 

**Published:** 1898-09

**Authors:** 


ARTICLE X.

### THE FIRST SUBSCRIBERS TO THE FIRST DENTAL JOURNAL.

At a recent meeting of the Odontographic Society, of Chicago, in a paper read before that body, some references was made to the origin of journalism in the dental profession.

The AMERICAN JOURNAL OF DENTAL SCIENCE was the first dental journal published in the world. Some reference was made in the discussion of the subject to the interest manifested by the members of the profession in this the first journalistic enterprise. Reference was made to the number of subscribers and the warm interest manifested by many of them in support of the journal. The journal was published quarterly at $5.00 per year. It will be seen by this list that many subscribed for more than one volume each, some taking forty copies, others twenty, ten, etc.

It was furthermore suggested that the names of these supporters of the first journal be placed upon permanent record, in as much as this list is now accessible to but very few in the profession.

Not one in five hundred of the practicing dentists of the country, perhaps, would know now where to find the names of these persons.

Dr. G. V. Black was the first to suggest the publication of these names, and doubtless many will look over them with more than an ordinary interest.

Ayers, Daniel, Amsterdam, Mont., County, N. Y. 1

Allen, Richard L., Saratoga Springs. 1

Alcock, James, N. Y. 2

Austin, George, Baltimore. 1

Austin, Nathan'l, Harrisonburg, Va. 2

Ash, Claudius, London. 2

Azling, Isaac, M D., Boston. 1

Auten, F. P., Lambertville, N. J. 1

Atkinson, John, Leeds, Eng. 1

Arthur, Robert, Baltimore. 1

Andrews, E. H., Charlotte C. H., N. Carolina. 1

Ash, G. E., London. 1

Arnold, Wm., M. D., N. Y. 1

Avery, Samuel, N. Orleans. 1

Brewster, C. S., Paris, France 1

Burdell, John, N. Y. 20

Bridges, M. K., Brooklyn. 20

Baker, Elisha, N. Y. 20

Brown, A. W., N. Y. 20

Bryan, Elijah, N. Y 1

Blake, Elihu, N. Y. 1

Bancroft, T. L., Granville, Ohio. 1

Brown, B. B., St. Louis, Mo. 2

Backus, G, Nashville, Tenn. 1

Briscoe, A. W., Baltimore. 1

Barstow, Wm. H., M. D., Georgetown, Ky. 1

Buchan, David L., Hempstead, Md. 1

Bidgood, Rich'd W., Smith, Va. 1

Badger, Felix H., Decatur, Ga. 1

Brown, Solyman, N. Y. 5

Fundenberg, G. B., Dentist, Pittsburgh, Pa. 1

Franklin, B. W., Fairfield, Herkimer, Co., N. Y. 1

Fraetas, J. A., New York. 20

Fay, Timothy, Baton Rouge, La. 1

Fay, Solomon, Chester Factories, Mass. 1

Ferguson, Jas. H., Northumberland C. H., Va. 1

Fouche, W. W., Philadelphia. 1

Falconer, John, New York. 1

Gilfiland, Doctor, Brooklyn. 1

Gaines, Rich. W., Charlotte C. H., Virginia. 1

Garrison, Doctor, Brooklyn. 1

Gallop, L. F., Newport, R. I. 1

Gardette, E. B., Philadelphia. 2

Green, L. T., Nashville, Tenn. 1

Goddard, W. H., Louisville. Ky. 1

Gill, Bryson, Baltimore. 1

Gunnell, Jas. S., M. D, Washington. 1

Griffith, S. & E., Louisville, Ky. 1

Geer, Rev. John A., Maryland. 1

Grandhomme, M. P., Paris, France. 1

Gidney, Eleazar, Man., Eng. 1

Greenwood, Isaac J., New York. 40

Grant, C. W., Newburg, New York. 1

Greenleaf, Chas., Hartford, Conn. 1

Becht, A. J., Hague, Netherlands. 1

Bareud, J. Dent., George St., Manchester. 1

Barend, Sam'l, Liverpool, Eng. 1

Bradford, D., Augusta, Ky. 1

Bell, Thomas, London. 1

Ballanger, D. W., Montgomery, Ala. 2

Brockway, Josephus, Troy. 2

Blanding, S., M. D., Columbia, S. C. 1

Baldwin, James Oscar, Newark, N. J. 1

Brown, Chas. D., Philadelphia. 1

Burdell, Harvey, M. D., New York. 2

Bliss, S., M. D., Syracuse, New York. 1

Buck, J. B., New York. 1

Briscoe, Jas. H., Phila. 1

Boyken, F. M., Smithfield, Va. 1

Burr, Hudson, Philadelphia. 1

Ballard, Geo. W., Madison, Morgan Co., Ga. 1

Bull, Abel, M. D., Boston. 1

Bemis, S A., Boston. 1

Budd, John D., Mount Holly, N. J. 1

Burr, Wm. H., Mount Holly, N. J. 1

Cook, Doctor, Brooklyn. 1

Clute, Nicholas, Louisville, Ky. 2

Chandler, James, Schenectady, N. Y. 1

Cobb, B. C., M. D., Clarkstore, Martins Co., N C. 1

Cox, A. L., M. D., New York. 1

Cleveland, J. A. Charleston, S. C. 1

Crocker, Fred'k, Sag Harbour, L. I. 1

Clark, F. H., Baltimore. 1

Comegys, —— Baltimore. 1

Cutler, Wm. Daily, Baltimore. 1

Cutler, Sam'l Jackson, Balmore. 1

Coleman, R. K., Baltimore. 1

Cauling & Edmunds, Baltimore. 1

Cobb, Anson, Brooklyn. 1

Clark, C., Jacksonville, Ill. 1

Clark, James, Springfield, Ill. 1

Ganson, Holton, Batavia, New York. 1

Gaines, B. B., Cassville, Ga. 1

Githens, John H., Phila. 1

Gunn, —— Nashville, Tenn. 1

Grimes, Doctor, Greenborough, Georgia. 1

Herd, Doctor, Brooklyn. 1

Harris, Chapin A., M. D., Baltimore. 40

Hawes, Arnold, Pawtucket, R. I. 1

Hulihen, J. P., Wheeling, W. Va. 1

Hawes & Allen, New York. 1

Houston, P., New York and Charleston. 1

Hartness, Thos. L., New York. 1

Hewlet, J. W., Greensboro, North Carolina. 1

Holmes, Oliver, Baltimore. 1

Howard, F., Washington, City. 1

Harris, John, M. D., Georgetown, Ky. 2

Hall, A. S., M. D., Scotland Neck, North Carolina. 1

Hubberd, E. R., Newbern, North Carolina. 1

Harper, Sam'l, Kent Island, Maryland. 1

Hand, G C., Easton, Pa. 1

Humphreys, Geo. W., Winchester, Virginia. 1

Hodgson, Doctor, Whiteplains, New York. 1

Harrison, R. H., M. D., Huntsville, Alabama. 1

Helsby, —— Manchester, England. 1

Hudson, Edward, Philadelphia. 1

Hamlin, T. B., Wythville, Virginia. 1

Hallified, Doctor, Petersburg, Virginia. 1

Hought, Chas. J., Philadelphia. 1

Hughes, H. W., Westminster, Maryland. 1

Hill, H., Norwalk, Conn. 1

Hoit, Emmett M., Stanwich, Connecticut. 1

Halleyman, W. F., Maytinton, South Carolina. 1

Imrie, —— Dentist, Manchester, England. 1

Cassill, J. F., Upper Marion Col., Mo. 1

Chewing, F. B., Rich., Va. 2

Clark, James, Lebanon, O. 1

Coppel, Chas., Preston, Eng. 1

Crane, O. P., Geneva, N. Y. 1

Chevalier, J. D., N. Y. 1

Crofoot, E. E., Middletown, Conn. 1

Crofoot, L. L., Middletown, Conn. 1

Chandee, J. G., Troy, N. Y. 1

Clark, A., Penyan, N. Y. 1

Cameron, James, Philadelphia. 1

Copeland, W. S., M. D., Rich Square, Va. 1

Carter, J. H., Ravenna, Portage Co., O. 1

Caldwell, Geo. H., Rushville, Ia. 1

Camp, W. C., Oxford, Granville, Co., North Carolina. 1

Cuyler, Vernon, M. D., Hartford, Conn. 1

Caldwell, D., Philadelphia. 1

Copelin, J., New York. 1

Crane, W. S., Hartford, Conn. 1

Culp, Clark, Philadelphia. 1

Dexter, Doctor, Brooklyn. 1

Dunning, H. H., Buffalo. 1

Davis, W. H. H., Cassville, Oneida Co., N. Y. 1

Duncan, Archibald, N. Y. 1

Davesson, Frederick A., M. D., Hillsboro, London Co., Va. 1

Dunlap, Joseph, Chillicothe, O. 1

Dunbar, J. R. H., M. D., Baltimore. 1

Desha, J. R., M. D., Little Rock, Ark. 1

De Loude, Le Chas., Wolverhampton, England. 1

Dodge, J. Smith, N. Y. 1

Dewar, Henry, Edinburgh, Scotland. 2

Dodge, Andrew, Matanzas, Cuba. 1

Dixon, Rufus E., M. D., Boston. 1

Doolittle, A. B., Plymouth, Conn. 1

Esterly, D., M. D., Troy, New York. 1

Ingraham, Thomas, Phila. 1

Johnson, Wm., Hagerstown, Maryland. 2

Jenks, W. D., Frederickstown, Maryland. 2

J'ollen, John N., York C. H., Virginia, 1

King, Doctor, Brooklyn. 1

Kelley, Elbridge G., Newburyport, Massachusetts. 2

Knower, Daniel, New York. 1

Keen, Benjamin F., Hills, Ga. 1

Knapp, F. H., Baltimore. 1

Kearsing, George, New York. 20

Kingsbury, C. A., Philadelphia. 1

Kimball, Horace, New York. 1

Keemy, B. M., Hudson, N. Y. 1

Koecker, Leonard, M. D., London. 2

Keene, Doctor, Newtown, Scott Co., Kentucky. 1

Latham, Hiram, Brooklyn. 1

Lum, John, Patterson, N. J. 1

Levett, M., New York. 1

Lovejoy, John, New York. 1

Laroque, Edward, Baltimore. 1

Lapham, B. B., Baltimore. 1

Lawrence, J., M. D., Tarboro, N. C. 1

Leadbetter, John, Alexan., D. C. 1

Lyon, S. K., New Orleans. 4

Llloyd, T. B., Manchester, England. 1

Lloyd, Rich., Liv., Eng. 1

Laird, O. P., Columbus, Ga. 1

Lamphire, Wm., Alexan., D. C. 1

Lawrence, S. W., Philadelphia. 1

Lee, Joseph, M. D., Camden, S. C. 1

Lawrence, E. B., Crawfordville, Ga. 1

Loomis, J. C., Carlisle, Pa. 1

Latimer, James, Madison, Morgan Co., Georgia. 1

Lethbridge, Sam'l, Richmond, Va. 1

Marvin, Doctor, Brooklyn. 1

Miller, Seth P., Worcester, Mass. 1

Maynard, Ewd., M. D., Washington City. 20

Milhau, John, New York. 1

Early, —— Doctor, Lynchburg, Va. 2

Elmendorf, Joseph, Penn-yan, N. Y. 1

Ellery, E., Baltimore. 1

Evans, J. N., M. D , Cynthiana, Ky. 1

Elliott, Joseph D., Leicester, Mass. I

Ensor, —— Dentist, Liverpool. 1

Ellis, Calvin, M. D., Boston. 1

Evans, G. W., Cincinnati. 1

Epps, W. J., M. D., Langhorne's P. O., Virginia. 1

Evans, Thos. W., Philadelphia. 1

Easton, Wm. T. Providence, R. I. 1

Fenn, Horatio N., M. D., Rochester. 2

Foster, J. H., M. D., New York. 2

Flagg, Josiah F. M., M. D., Boston. 1

Frazer, Doctor, Cynthiana, Harrison, Co., Ky. 1

Follen, John H., North C. H., Va. 1

Frink, J. N., Portland, N. H. 1

Foote, George, Vernon, N. Y. 1

Faulkner & Pierpont, Man., England. 1

Martin, Chas. F., Norfolk, Va. 1

M'Kinney, W. N., Fredricksburg, Va. 20

Munson, W. G., New Haven. 1

Manly, Horace, Canada, N. Y. 1

Macall, Leonard, M. D., Baltimore. 1

Miller, J. H., Professor, etc., Baltimore. 1

Merryman, Geo., Baltimore. 1

Manning, M. E., Tarboro, North Carolina. 2

M'Cabe, James D., Richmond, Va. 2

McDonald, G., M. D., Macon, Georgia. 2

Macey, Wm. M., M. D., White Sulphur, Scott Co., Kentucky. 1

McAllister, J. M., Albany. 1

McNaughton, M. A., Albany. 1

Mason, Doctor, Brooklyn. 1

Merritt, C., Bridgeport, Con. 1

Martin, J., Portsmouth, Eng. 1

MacPherson, James, Glasgow, Scot. 1

Middleton, Ellis, Philadelphia. 1

McIlhinney, Joseph E., Philadelphia. 1

Matson, Alpheus, Auburn, New York. 1

M'Grath, R., Philadelphia. 1

More, Justus E., Philadelphia. 1

Middleton, Wm., New York. 1

Merritt, Chas., Bridgeport, Conn. 1

Murrill, L., Petersburg, Va. 1

Nelson, Alexander, Albany. 1

Norman, S. P., Little Rock, Arkansas. 4

Neal, D., Philadelphia. 1

Noyes, Enoch, Baltimore. 20

Nasmyth, Robert, Edinburgh, Scotland. 1

Overfield, M., Winchester, Virginia. 2

Parker, Doctor, Brooklyn. 1

Parmly, L. S., New Orleans. 20

Parmly, Jahial, New York. 20

Parmly, Jahial. Savannah. 1

Parmly, Geo. W., N. Orleans. 1

Parmly, David, New York. 1

Parmly, Eleazar, New York. 40

Parmly, Rudolph, Mobile. 1

Parmly, W. Sam'l, N. York. 1

Patello, Wm. H., Charlotte C. H., Virginia. 1

Park, David N., A. M., New York. 1

Parkhurst, Wm. H., N. Y. 1

Peak, J. M., Cooperstown, New York. 1

Plant, Ebenezer, Willimantic, Conn. 1

Pritchard, P. C. Jackson, North Carolina. 1

Plough, A. L., New Orleans. 1

Parsons, J. H., Liverpool, England. 1

Palmer, W. A., Stratford, Connecticut. 1

Pleasants, Chas., Plainesville, O. 1

Perkins, Jacob, Springfield, Mass. 1

Parker, T. H., Philadelphia. 1

Pent, George, M. D., Lawrenceville, Virginia. 1

Pancost, S., Shelbyville, Ky. 1

Royce, W. A., Poughkeepsie, New York. 3

Robinson, E. C., Norfolk, Va. 1

Roper, Lewis, M. D., Philadelphia. 20

Rowell, Chas., New York. 2

Roe, Early, M. D., Hillsborough, Ga. 1

Rodriguez, B. A., M. D., Charles, S. C. 1

Ritter, —— Washington City. 1

Robinson, E., M. D., Leesburg, Ky. 1

Reynolds, Wm., M. D., Camden, S. C. 1

Reinstein, Frederick, Philadelphia. 1

Ross, Samuel, New York. 1

Root, J. B., Hamilton, Madison Co., New York 1

Reese, F. A., M. D., Hamilton, Bermuda. 1

Strickland, Benj., Cleveland, O. 1

Snow, R. J., Buffalo. 1

Smith & Thackston, Farmville, Va. 1

Scott, W. R., Raleigh, N. C. 4

Shuff, P. L., M. D., Leesburg, Ky. 1

Sneeds, M. D., Frankfort, Kentucky. 1

Stringfellow, S. L., Balto. 20

Stratton, Chas., Keene, N. H. 1

Sanders, M., London. 1

Sanborn, E., Andover, Mass. 1

Smith, Wm , Liverpool, Eng. 1

Stinkley, B. D., Albany. 1

Smith, G. W., New Orleans. 1

Stuart, R., Knoxville, E. T. 1

Smith, J. W., Amherst, Mass. 1

Searle, F., Springfield, Mass. 1

Stockton, S. W., Philadelphia. 20

Stowell, John, Philadelphia. 1

Shepherd, S. M., Petersburg, Va. 1

Sherman, A., Newark, N. J. 2

Simpson, ——, Manchester, England. 1

Sale, T. A., Williamsboro, North Carolina. 1

Sims, J. M., M. D , Fish Dam, Union Dis., S. C. 1

Sanders, E., 16 Argyle st., London. 1

Stevens, B. H., Elbridge, Onondaga Co., N. Y. 1

Taylor, Edward, M. D., Bainbridge, O. 2

Tucker, E. G., M. D., New York. 2

Trenor, John, M. D., New York. 1

Trenor, James, M. D., N. Y. 1

Tilyard, H. W., Baltimore. 1

Taliaferro, T. S., M. D., Maysville, Ky. 1

Thompson, ——, Doctor, Columbus, O. 1

Thorn, Doctor, Brooklyn. 1

Tyler, Nathaniel, Chicapee Falls, Mass. 1

Taylor, James, Crawfordville, Ia. 1

Teter, G. T., Dentist, Greenfield, Highland Co., Ohio. 1

Thorn, Doctor, Edgefield, C. H., South Carolina. 1

Thackston, Doctor, Farmville, Va. 1

Townsend, Sam'l, Baltimore. 1

Truman. Geo., Philadelphia. 1

Van Praag, A. S., New York. 1

Vincent, Ezra, New York. 1

Van Camp, Louisville, Ky. 1

Van Paten, C. H., Pittsburg. 1

Vanboskirk, L. E., St. John, N. B. 1

Willard, M. T.. Concord, New Hampshire. 1

Weed, J. Sacket, W. Greenfield, N. Y. 1

White, Geo. H., New York. 1

Walker & Jones, New York. 20

Wanzer, N. C., Auburn, New York. 1

Wayt, John G., Richmond, Virginia. 2

Wilson, J. D., Richmond, Virginia. 2

Ware, W., M. D., Wilmington, N. C. 1

Wheat, J. B., New Haven. 1

White, S. D., Philadelphia. 1

Worthington, R. C., M. D., Murfreesboro, N. C. 1

Ward, David G., Wanesboro, N. C. 1

Ward, W. A., Petersburg, Va. 2

Willmore, E. Baltimore. 1

Wheeler, E. D., Hillsboro, Coffee Co., Tenn. 1

Williams, E. C., M. D., Philadelphia. 1

Wells, H., Hartford, Conn. 1

Young. H., Troy, N. Y. 1

Yard, George Philadelphia. 1

### To Readers and Correspondents.

Communications intended for publication, and exchange journals and papers, should be addressed to the Editor of The American Journal of Dental Science, No. 845 N. Eutaw St. Baltimore, Md. All letters relating to the business of the Journal, or containing cash remittances, to be directed to Snowden & Cowman Mfg. Co., 9 W. Fayette Street, Baltimore, Md. Articles for publication should be received by the Editor at least two weeks previous to the first of the month on which the number in which it is designed for them to appear is issued.

### CONTENTS:

### SELECTIONS.

☞ The American Journal of Dental Science will contain fifty pages of reading matter in each number, making a volume of about Six Hundred pages,

EXCLUSIVE OF ADVERTISEMENTS.

